# Affordable Care Act Medicaid expansion, access to health care, and financial behavior of the United States adults

**DOI:** 10.1057/s41271-024-00522-0

**Published:** 2024-09-23

**Authors:** Redwan Bin Abdul Baten, Abdullah Noman, Mohammad Nakibur Rahman

**Affiliations:** 1 Department of Health Management and Policy, College of Health and Human Services, University of North Carolina at Charlotte, 9201 University City Blvd., Charlotte, NC 28223 USA; 2 Thomas College of Business and Economics, University of North Carolina at Pembroke, Pembroke, NC 28372 USA

**Keywords:** Medicaid expansion, Access to care, Healthcare coverage, Financial behavior, Financial preparedness, Social determinants of health

## Abstract

The access to care benefits of Affordable Care Act (ACA) Medicaid expansions are important for 45–64-year-old adults who are living below 100% of the Federal Poverty Level, a particularly vulnerable group in the United States (US). Gaining coverage from Medicaid expansions should improve access to healthcare and affect social determinants of health, including financial behavior. We analyzed data from 2009 to 2018 from the National Financial Capability Survey (NFCS) and utilize a difference-in-differences model to compare outcomes changes in states with and without expansion before and after the ACA Medicaid expansions. Overall, Medicaid expansion was associated with increased healthcare access for 45–64-year-olds, potentially resulting in better healthcare experience. Results indicate effects of the Medicaid expansion on the financial behavior of 45–64-year-olds, with evidence of credit card bills being paid in full, higher banking activities, and better financial preparedness. These findings have important implications for financial regulators and healthcare policymakers.

**Supplementary Information:**

The online version contains supplementary material available at 10.1057/s41271-024-00522-0.

## Key messages

- The 2010 Affordable Care Act (ACA) Medicaid expansions aimed to increase coverage for vulnerable populations, including low-income 45–64-year-olds with worse health status and nearing retirement.
- Medicaid expansion increased access to care for 45–64-year-olds and had potential effects on their financial behavior, including improvements in credit cards being paid in full, higher banking activities, and better financial preparedness.
- Our findings demonstrate that health policies such as Medicaid expansion can have effects on important social determinants of health, such as financial behaviors.

## Introduction

The Affordable Care Act (ACA) of 2010 increased Medicaid eligibility to 138% of the Federal Poverty Level (FPL) for adults younger than 65 in states that expanded Medicaid under the ACA. By 2023, 40 states and Washington, DC, have expanded Medicaid under the ACA [1]. Before this expansion, Medicaid income eligibility varied across states but was overall much lower than the ACA Medicaid expansion limit. The Medicaid expansions significantly increased coverage and access to care for low-income individuals in expansion states [2–6] and improved health status [7–9]. Studies have found increases in Medicaid coverage and a drop in the uninsured rate, [2, 10–13] better self-rated health status [7], improvement in activities of daily living [14], reduction in any work limitations from health [15], and reduction in disease-related deaths [16] for the 50–64-year-old age group.

In the United States, adults within a few years of reaching 65 with low income are a particularly vulnerable age group. Due to low Medicaid eligibility levels and greater difficulty in accessing private coverage than higher-income adults, this age group has had high uninsured rates historically [17]. They are less able to purchase coverage independently, less likely to have employer-sponsored coverage due to less skilled jobs [18], have part-time employment or higher unemployment rates, and are not yet eligible for Medicare, which begins at age 65. They are at a greater risk of experiencing significant health events and incurring substantial out-of-pocket medical expenses than same-age, higher-income individuals [17]. In 2013, individuals aged 55–64 had the highest annual out-of-pocket spending on healthcare exceeding $2,000 [12]. Compared to younger adults, this age group has more unmet healthcare needs, and a higher prevalence of chronic conditions [6, 19–21]. Between 2011 and 2019, 65% of 50–64-year-olds reported at least one chronic condition, while 35% reported having two or more conditions, which was higher than those aged 40–49 [22]. To detect and manage chronic health conditions, access to primary care is thus vital for this age group. 45–64-year-old low-income adults may benefit more in access and health gains from Medicaid expansions than younger adults.

45–64-year-olds must make retirement decisions that are partly dependent on the available health insurance coverage options, which could adversely affect health and reduce financial security [23]. Compared to 18–49-year-olds, 50–64-year-olds have more concerns about having money to live comfortably after retirement [24]. Between 2007 and 2011, households headed by 55–64-year-olds experienced a decline in median wealth of about $72,000 [25]. Those aged over 50 are more likely to carry debt and are more indebted than previous generations [26]. The debt-to-net-wealth ratio of 50–64-year-olds increased from 16% in 1992 to 27% in 2016 [27]. Adults aged 50 or more have 22% of more than $1.5 trillion in student loans, representing 7% of their total debts [28]. When faced with hospitalizations, non-elderly adults face considerable financial risks, even after having insurance [29].

Recent studies have captured the effects of the ACA beyond healthcare on various Social Determinants of Health (SDOH) [30]. As secondary effects, Medicaid expansion has decreased high school dropout rates [31], improved outcomes for justice-involved individuals [32, 33] and those experiencing homelessness [34], reductions in crime rates [35], decreased food insecurity [36] and home evictions [37], increased utilization of pediatric preventive care [38] and dental insurance coverage [39], etc.

Several studies have found the ACA to have indirect effects on the financial aspects of a beneficiary’s life [40]. Recent studies have established financial capability as an important SDOH [41]. Evidence from pre-ACA policy changes, such as the Oregon Medicaid experiment, demonstrates that Medicaid expansion led to stabilized incomes and greater financial stability [42]. ACA Medicaid expansions have improved financial behaviors such as—a reduction in unpaid medical bills [40, 43, 44], prevention of new delinquencies [40], improved credit scores [40], increased available credits [40], reduced number of debts sent to third parties [43], reduction in collection balances [43], improvement in satisfaction with one’s financial situation [45], reduction in catastrophic health expenditure risk [46], and poverty rates [47]. Another study found improvements in public records such as eviction, judgment, and bankruptcies [44]. Medicaid expansion effects on financial health were more prominent among those with greater medical needs, such as those with chronic illnesses [44], which are highly prevalent in 45–64-year-olds.

Financial behavior is an important predictor of post-retirement financial well-being and may include several components, such as financial preparedness, financial literacy, the ability to navigate financial products such as credit cards, the baking system, retirement investments, etc. [48]. Financial knowledge or literacy significantly impacts future financial outcomes, especially for older individuals [49]. Individual retirement plans affect savings, consumption, and investment decisions and, therefore, have important implications for the well-being of older workers. This is important because financial literacy and behavior may take time to improve [50]. Furthermore, the greater financial security associated with gaining health insurance can reduce stress and increase resources to meet other basic needs such as nutrition or housing. Financial preparedness is thus essential for 45–64-year-olds as individuals considering early retirement decisions, which are often closely associated with the health of individuals [51]. The ACA provisions intended to increase access to affordable insurance not tied to employers have resulted in improved or flexible retirement decisions among those without employer-sponsored insurance [52]. The ACA Medicaid expansions thus give us a unique opportunity to study financial behavioral outcomes utilizing several years of post-expansion data.

To the best of our knowledge, previous studies have not analyzed the effects of Medicaid expansion on financial behavior. Some of these financial effects have been documented for broader age groups of non-older adults and not specifically for individuals aged 45–64. We utilize a nationally representative data set with 4 years of post-expansion data (2014–2018) to understand the impacts on financial behavior. Utilizing a quasi-experimental model, we provide the first empirical evidence of changes in financial behavior, including financial preparedness from the ACA Medicaid expansions for the low-income 45–64 age group. Our study sheds light on the implications of continuing disparity in access to healthcare and financial behavior for this age group.

Financial behavior is related to actions that reflect individuals’ attitudes to money and wealth in general. According to the framework developed by the Organization for Economic Cooperation Development (OECD), financial behavior includes paying bills on time, shopping habits, etc. [53]. Financial behavior is of two types: routine tasks and advanced tasks [48]. Routine tasks include making monthly payments, mortgage payments, and being able to buy basic food needs. Studies find that women with higher financial self-efficacy are more likely to hold investment and savings products and less likely to hold debt-related products [54].

An increase in income-based Medicaid eligibility can improve access to healthcare of adults aged 45–64 who gain coverage. Before the ACA expansions, many adults in this age group did not enjoy health benefits, forewent needed services and preventive care, and incurred higher medical bills and out-of-pocket expenditures. Therefore, we hypothesize that gaining Medicaid from the ACA expansions will increase access to health care by reducing out-of-pocket expenditures, which would reduce unpaid medical bills.

Similarly, the ACA Medicaid expansion can improve financial outcomes through several pathways. Medicaid expansion has health effects by improving health status [13], resulting in lower out-of-pocket [55] and medical expenditure [56], medical debt [57], unpaid medical bills [40]. A secondary effect of Medicaid expansion could result in increased disposable income for low-income beneficiaries [58, 59]. Medicaid expansion also plays a protective role for beneficiaries who have more social needs [60] by improving social determinants of health (SDOH), including housing and food security [61]. Through these pathways, Medicaid expansion has the potential to improve financial outcomes for the vulnerable low-income 45–64-year-old population.

We hypothesize that increased access would improve the financial behavior of older individuals with better management of their finances in the areas of credit cards, banking, and financial preparedness. This improvement might happen gradually for some individuals, or immediately for others if it addresses previously unaddressed financial problems.

## Data and methods

### Data sources and study sample

We use data from the National Financial Capability Study (NFCS) from 2009 to 2018 [62]. NFCS collects data on indicators of financial capability, including financial knowledge, resources, access, experience, attitudes, healthcare access, and socioeconomic and demographic characteristics. The FInancial Industry Regulatory Authority (FINRA) Investor Education Foundation commissioned the survey in 2009 to help policymakers understand the financial circumstances and needs of households across the US. From 2009, NFCS conducts an online survey every 3 years on a large nationally representative sample of over 25,000 American adults with 500 respondents in each of the 50 states and the District of Columbia.

As part of the study sample, we include adults aged 45–64 with household income below 100% of the Federal Poverty Level (FPL). We exclude individuals between 100 and 138% of the FPL from the main sample because they were eligible for subsidies in the ACA private insurance marketplace in non-expansion states, but not in expansion states [63]. To demonstrate the unique characteristics of the 45–64-year-old age group, we analyze and compare the outcomes with 18–44 and 65 + -year-olds separately.

We use the midpoints of the household income categories reported in NFCS to calculate household income as a percentage of FPL, considering family size and annual changes in FPL [13, 64]. Because NFCS did not ask individuals about the number of household members, we approximate that number based on the number of adults and children in the household. Living arrangement and marital status are considered to calculate the number of adults in the household (one for single or divorced/separated/widowed, two adults for married or living with spouse or parent, and three if living with parents). Because NFCS data is available from 2009 onwards at a 3-year interval, we start the sample period in 2009 and include data for 2012, 2015, and 2018. A descriptive analysis of the study sample is given as Supplemental Information.

We include all 50 states and Washington D.C. in the sample and consider the first full year of Medicaid expansion as the beginning of treatment status for that state [1]. States that never expanded Medicaid under the ACA are deemed control states. Therefore, we have distinct post-expansion years for all Medicaid expansion states as different states expanded their Medicaid program in unique years. The details of state assignments into treatment or control groups are given as Supplemental Information.

### Outcome measures

We utilize two measures for access to healthcare that have been commonly studied with the Medicaid expansions. These are binary indicators for (i) having any healthcare coverage and (ii) having unpaid bills from a healthcare or medical service provider that are due.

We analyze eight financial behavior outcomes and categorize them into three groups—credit card, banking, and financial preparedness. Credit card indicators include—(i) the number of credit cards and (ii) always paid credit card charges in full in the past 12 months.

We include three measures of banking. The first measure is a binary indicator of (iii) having a checking account. The second binary indicator is (iv) having a savings account, money market account, or Certificates of Deposit (CD). The last measure is (v) having any stocks, bonds, mutual funds, or other securities investments (not including retirement accounts).

We also include three measures of financial preparedness. The first indicator is (vi) satisfaction with one’s current personal financial condition, considering one’s assets, debts, and savings (10-point scale, where 10 is extremely satisfied). The second is a binary indicator of (vii) setting aside emergency or rainy-day funds to cover expenses for 3 months in case of sickness, job loss, economic downturn, or other emergencies. The last is a binary indicator for (viii) ever trying to figure out how much one needs to save for retirement.

### Study design and estimation

We applied a generalized difference-in-differences (DD) model to compare outcome changes within expansion or treatment states before and after the ACA Medicaid expansions to outcome changes within the non-expansion or control states. The DD regression model is specified as follows:

1 $$Y_{{{\text{ist}}}} = \beta_{0} + \beta_{1} \left( {{\text{Medicaid}}_{{{\text{st}}}} } \right) + \beta_{2} X_{{{\text{ist}}}} + \theta_{{\text{s}}} + \delta_{{\text{t}}} + \varepsilon_{{{\text{ist}}}} $$

where *Y*_ist_ is one of the healthcare access or financial behavioral outcomes mentioned above for an individual *i* in a state *s* in a year *t*; *Medicaid*_*st*_ is a variable that continuously captures the Medicaid expansion status of states over the years. *Medicaid*_*st*_ are equal to 1 for states when they expanded Medicaid under the ACA and 0 for when expansion did not occur. Hence, non-expansion or control states will always have *Medicaid*_*st*_ equal to 0 across all study years, whereas expansion states will have 0 only for the years that they did not expand their Medicaid program under the ACA. *X*_*ist*_ includes the following individual-level covariates: age, sex, race, education, marital status, whether there are children at home, employment status, and home ownership. θ_*s*_ represents state fixed effects (0/1 indicators), which capture time-invariant differences between states. δ_*t*_ represents year-fixed effects, which represent national trends shared between states. β_1_ is the difference-in-differences estimate of the Medicaid expansion effect on outcome *Y*.

The model (Eq. 1) assumes a flexible effect of the Medicaid expansions over time, such as the effect differs each year since enactment. To evaluate whether the Medicaid expansion effects change over time after the expansion, we estimate a second event study model. Due to the 3-year interval in the dataset, it was not possible to have a straightforward indicator for post-expansion year 1, year 2, etc. While most states expanded Medicaid in 2014, a smaller number of states expanded Medicaid in 2015 and 2016. However, NFCS did not publish data for 2014 and 2016. After considering these factors, we decided to estimate the effects for states that experienced the treatment or Medicaid expansion effects for different periods following previous studies [2, 3, 65–67]. This model is specified as follows:

2 $$Y_{{{\text{ist}}}} = \alpha_{0} + \alpha_{1} \left( {{\text{Medicaid}}_{{{\text{s1}}}} \times {\text{POST }}_{{\text{t}}} } \right) + \alpha_{2} \left( {{\text{Medicaid}}_{{{\text{s2}}}} \times {\text{POST}}_{{\text{t}}} } \right) + \alpha_{3} \left( {{\text{Medicaid}}_{{{\text{s3}}}} \times {\text{POST }}_{{\text{t}}} } \right) + \alpha_{4} X_{{{\text{ist}}}} + \theta_{{\text{s}}} + \delta_{{\text{t}}} + \varepsilon_{{{\text{ist}}}}$$

In this event study model, POST_t_ is a binary variable indicating the year in which states received the treatment or expanded Medicaid; Medicaid_s1_ indicates the two states (Louisiana and Montana) that expanded Medicaid in 2016 and experienced expansion for 2 years. Medicaid_s2_ indicates three states (Alaska, Indiana, and Pennsylvania) that expanded Medicaid in 2015 and experienced expansion for 3 years. Medicaid_s3_ includes 21 states that expanded in 2014 and experienced expansion for 4 years. α_1_ is the Medicaid expansion effect for states experiencing expansion for 2 years, while α_2_ and α_3_ are the expansion effects for 3 years and 4 years, respectively. This model provides all estimates relative to the pre-expansion period before 2014. All other variables are as defined previously.

The DD design assumes that outcomes would have changed similarly between the expansion and non-expansion states in 2014–2018 had the expansion not happened. To check this assumption, we follow previous literature [13, 64, 68] and compare outcome trends between expansion and non-expansion states before the expansion year of 2014 using a regression model like the above but limiting the data to 2009–2015. We then interact the Medicaid expansion indicator (Medicaid_st_) with one binary indicator for 2009, and another for 2012, with 2015 as the reference year. We include 2015 as the reference year due to limitations in the availability of the data for 2013 or 2014. We then test these interactions, whose significance, would indicate differential pre-trends that might bias the DD estimates.

We estimated the regression models using weighted least squares with the NFCS individual sampling weights. Standard errors are clustered at the state level. All analyses were conducted using Stata 17.

## Results

### Medicaid expansion effects on access to care

Table 1 reports the estimated ACA Medicaid expansion effects over 2014–2018 on access to care from Eq. 1. The estimates are for 45–64 years old adults below 100% of FPL. Relative to non-expansion states, expansion states had an increase in coverage compared to non-expansion states by 12.7 points (*p* < 0.01). Following Medicaid expansion, the likelihood of having unpaid medical bills in the past 12 months declined significantly by 5.9 percentage points (*p* < 0.10) in expansion states compared to non-expansion states. The ACA Medicaid expansion significantly increased coverage for 18–44-year-olds but not the 65 + and unpaid medical bills did not change for either age group.

**Table 1:** Difference-in-differences estimates of the ACA Medicaid expansion effects on the likelihood of health care access for adults aged 45–64 below 100% FPL, NFCS 2009–2018

	Effect of the ACA Medicaid expansion 2014–2018		
	45–64	18–44	65 +
Health Insurance Coverage	0.127*** (0.033)	0.109*** (0.025)	− 0.010 (0.032)
Unpaid Medical Bills	− 0.059*(0.034)	0.002 (0.024)	0.036 (0.066)

The models adjust for age, sex, race, education, marital status, children at home, employment status, and home ownership and include fixed effects for survey year and state. Standard errors are clustered by state and are presented in parentheses. The sample size ranges between 4400 and 5433, depending on the outcome. NFCS sampling weights are used

*ACA* Affordable Care Act, *NFCS* national financial capability study, *FPL* federal poverty level

*p < 0.10, **p < 0.05, ***p < 0.01

### Medicaid expansion effects on financial behavior

Table 2 presents the estimates of Medicaid expansion effects in 2014–2018 on the financial capabilities of adults aged 45–64 below 100% of FPL (Eq. 1). For credit card outcomes, there were no significant differences between Medicaid expansion and non-expansion states in terms of the number of credit cards that the respondents had. However, there was a significant improvement in the probability of paying credit card charges in full by 8.6 percentage points (*p* < 0.10).

**Table 2:** Difference-in-differences estimates of the ACA Medicaid expansion effects on the likelihood of financial behaviors for adults aged 45–64 below 100% FPL, NFCS 2009–2018

	Effect of the ACA Medicaid Expansion 2014–2018		
	45–64	18–44	65 +
Credit card			
Number of credit cards	0.009 (0.039)	− 0.053** (0.022)	0.044 (0.059)
Paid credit cards in full	0.086* (0.044)	0.011 (0.032)	− 0.039 (0.069)
Bank			
Has a checking account	0.025 (0.029)	− 0.011 (0.020)	0.028 (0.038)
Has a savings account, money market account, or CDs	0.018 (0.031)	− 0.022 (0.022)	− 0.035 (0.066)
Has investments in stocks, bonds, mutual funds, or other securities	0.003 (0.017)	0.019 (0.018)	− 0.044 (0.034)
Financial preparedness			
Satisfied with your current personal financial condition	0.120 (0.143)	0.140 (0.105)	− 0.554* (0.315)
Set aside emergency or rainy-day funds	0.048** (0.023)	0.032 (0.020)	− 0.020 (0.042)
Tried to figure out how much is needed to save for retirement	0.040 (0.032)	− 0.004 (0.017)	− 0.049 (0.124)

The models adjust for age, sex, race, education, marital status, children at home, employment status, and home ownership and include fixed effects for survey year and state. Standard errors are clustered by state and are presented in parentheses. The sample size ranges between 2222 and 5403, depending on the outcome. NFCS sampling weights are used

*ACA* Affordable Care Act, *NFCS* national financial capability study, *FPL* federal poverty level

*p < 0.10, **p < 0.05, ***p < 0.01

For banking outcomes, there was suggestive evidence of improvements in the number of checking accounts, savings accounts, and having investments in stocks/bonds/mutual funds or other securities for 45–64-year-olds. However, the difference between expansion states and non-expansion states was not statistically significant.

For financial preparedness outcomes, setting aside emergency or rainy-day funds increased significantly by 4.8 percentage points (*p* < 0.05). There was suggestive evidence of increased levels of trying to figure out how much to save for retirement and increased levels of satisfaction with one’s current personal financial condition, but the results were not significantly different between expansion and non-expansion states. For all financial behavior outcomes, the results were different in magnitude or direction for 18–44 and 65 + -year-olds, demonstrating the unique circumstances of the 45–64-year-old age group.

### Event study results

Table 3 reports the Medicaid expansion effect estimates separately for states experiencing 2, 3, and 4 years of expansion relative to the pre-expansion period (Eq. 2). Overall, access to care effects were comparable between the three groups of states for coverage and unpaid medical bills. However, states experiencing expansion for 4 years had an 8.6 percentage point (p < 0.10) increase in health insurance coverage, whereas the results were not significant for states having 2 or 3 years of expansion. Improvements in unpaid medical bills were significantly different by 10 percentage points (p < 0.01) between Medicaid expansion and non-expansion states for states experiencing 2 years of expansion. The estimates were in a similar direction but insignificant for states with 3 or 4 years of expansion.

**Table 3:** Year-by-year difference-in-differences estimates of the ACA Medicaid expansion effects on the likelihood of health care access for adults aged 45–64 below 100% FPL, NFCS 2009–2018

	Effect of the ACA Medicaid Expansion 2014–2018		
	2 Years of Expansion	3 Years of Expansion	4 Years of Expansion
Health insurance coverage	0.129 (0.092)	0.004 (0.089)	0.086* (0.050)
Unpaid medical bills	− 0.100*** (0.028)	− 0.003 (0.079)	− 0.051 (0.037)

The models adjust for age, sex, race, education, marital status, children at home, employment status, and home ownership and include fixed effects for survey year and state. Standard errors are clustered by state and are presented in parentheses. The sample size ranges between 4400 and 5433, depending on the outcome. NFCS sampling weights are used

*ACA* Affordable Care Act, *NFCS* national financial capability study, *FPL* federal poverty level

*p < 0.10, **p < 0.05, ***p < 0.01

Table 4 reports the estimates of Medicaid expansion effects on financial behavioral outcomes separately for states experiencing 2, 3, or 4 years of expansion with the pre-expansion years as the reference period. Paying credit cards in full was larger and statistically significant for states with 3 years of expansion 23.7 percentage points (*p* < 0.05), with suggestive improvements for states with 2 and 4 years of expansion. For states with 2 years of expansion, there was a 9.8 percentage point (*p* < 0.10) increase in the likelihood of having a checking account and a 14.5-percentage point (*p* < 0.01) increase in the likelihood of having a savings account, money market, or CDs. Setting aside emergency or rainy-day funds improved significantly by 17.6 percentage points (*p* < 0.01) in 2021. Trying to figure out how much is needed to save for retirement increased by 17.6 percentage points (*p* < 0.01) for states with 2 years of expansion but was not significant for states with longer duration of expansion. Differences in estimates for satisfaction with one’s current personal financial condition improved significantly by 0.86 points (p < 0.01) on a 10-point scale.

**Table 4:** Year-by-year difference-in-differences estimates of the ACA Medicaid expansion effects on the likelihood of financial behaviors for adults aged 45–64 below 100% FPL, NFCS 2009–2018

	Effect of the ACA Medicaid expansion 2014–2018		
	2 Years of expansion	3 Years of expansion	4 Years of expansion
Credit card			
Number of credit cards	− 0.120** (0.045)	0.085 (0.067)	− 0.050 (0.040)
Paid credit cards in full	0.073 (0.083)	0.237** (0.090)	0.014 (0.056)
Bank			
Has a checking account	0.098* (0.055)	− 0.042 (0.079)	0.022 (0.042)
Has a savings account, money market account, or CDs	0.145*** (0.036)	0.056 (0.073)	0.009 (0.044)
Has investments in stocks, bonds, mutual funds, or other securities	− 0.015 (0.019)	− 0.012 (0.035)	− 0.001 (0.025)
Financial preparedness			
Satisfied with your current personal financial condition	0.858*** (0.198)	0.494 (0.402)	− 0.013 (0.176)
Set aside emergency or rainy-day funds	0.176*** (0.055)	0.021 (0.061)	0.022 (0.028)
Tried to figure out how much is needed to save for retirement	− 0.039 (0.183)	− 0.035 (0.064)	0.032 (0.027)

The models adjust for age, sex, race, education, marital status, children at home, employment status, and home ownership and include fixed effects for survey year and state. Standard errors are clustered by state and are presented in parentheses. The sample size ranges between 2222 and 5403, depending on the outcome. NFCS sampling weights are used

*p < 0.10, **p < 0.05, ***p < 0.01

*ACA* Affordable Care Act, *NFCS* national financial capability study, *FPL* federal poverty level

### Robustness checks

We perform five robustness checks to check the sensitivity of our results. In the first robustness check, we include 45–64-year-old individuals below 138% of FPL. A second test includes in the sample the same age group from 138 to 400% of FPL. In another robustness check, we test if the effects of Medicaid expansion were prevalent among those above 400% of FPL. We test the robustness of our estimates by analyzing a model excluding survey weights. The last robustness checks included 2021 data in the sample. The results from these analyses are generally similar to the main results for access (Table S3) and financial (Table S4) outcomes. The results of the robustness models confirm the validity of our study estimates.

### Pre-expansion trend checks

As noted above, we compare outcome trends between expansion and non-expansion states before 2014 as a check of the difference-in-differences design. Table S5 reports the estimates of pre-expansion trends for access to care by Medicaid expansion status. There are no statistically significant differences between both access outcomes between expansion and non-expansion states. Overall, these results support the validity of the difference-in-differences estimates for the access to care measures.

Table S6 reports the pre-trends of financial behavior measures by Medicaid expansion status. No statistically significant differences were observed between expansion and non-expansion states in pre-trends of credit card or financial preparedness outcomes (based on joint year tests or individual year differences). Overall, these results support the robustness of the estimated effects of the Medicaid expansions on financial behavioral outcomes.

## Discussion

This paper contributes to the growing literature addressing the interrelationship between health and finance. Our results indicate that following the Medicaid expansion, the most positive changes have been increased health insurance coverage for adults aged 45–64 below 100% FPL. We find coverage improvements by 12.7 percentage points, which is similar to another study that found a 15-percentage point increase in coverage for 50–64-year-olds [14]. This is very important for people with low income and poor general health as they tend to lack preparedness for their retirement life and often make financial decisions that are myopic in nature [51]. Similarly, households with poor health status tend to hold a limited number of relatively safer assets in their investment portfolio [69]. This could potentially deprive these households of the upside market gains that would come from relatively riskier assets.

The event study shows that certain financial behavioral indicators improve a few years after expansion. Medicaid has certain long-term benefits that are evident from longitudinal studies. Studies have found long-term impacts of the Medicaid program as it shielded Medicaid beneficiaries from growing out-of-pocket expenditures [47, 70]. Similarly, important SDOH indicators such as financial behavioral outcomes might improve over time for various reasons [50].

By 2030, the proportion of people in the US over 65 will exceed 20% [71]. People aged 65 and older have a greater need for assistance in all affairs, including healthcare and financial decisions. Therefore, it is important for individuals to prepare for their life after retirement. Studies have highlighted the importance of research on the intersection between household finance and health situation [72]. Against this backdrop, our study finds that Medicaid expansion is associated with increased healthcare coverage, evidence of credit card debt being paid in full, higher banking activities, and better financial preparedness for the future. Our findings are supported by other studies, which show that the financial behavior of individuals has important implications for financial well-being and life satisfaction [73].

### Limitations

Our study has several limitations. First, not having data for every year throughout the study period has limited the sample size and the ability to perform a year-by-year event study. Second, we could not perform sub-group analysis as the small sample size would be prohibitive in gaining meaningful results. Third, our analysis could not account for the study participants’ financial records or health status, including chronic conditions. Fourth, our study utilizes the generalized DD model, which has various limitations [74]. However, due to the intermittent nature of the NFCS dataset, the CSDID method could not be performed as a robustness check as suggested by Callaway and Sant’Anna (2024). Due to the data limitations, we had to use 2015 as the reference period in the pre-trend checks, where ideally the pre-trend model would be restricted to the pre-treatment period.

## Conclusions

This paper explores the potential effects of Medicaid expansion on the personal financial and healthcare experience of 45–64-year-olds. It focuses on a specific segment of the population aged between 45 and 64 below 100% of FPL. Results suggest that coverage expansions have improved access to care, and financial behavior for individuals aged 45–64. Findings from this study highlight the importance of continuing to evaluate the effects of Medicaid insurance expansions as the effects on various SDOHs may be evident in the long term. The empirical findings inform financial regulators and healthcare policymakers to continue to improve access to care among 45–64-year-old adults, as enhanced coverage would improve numerous SDOHs, including financial behaviors.

## Supplementary Information

Below is the link to the electronic supplementary material.

Supplementary file1 (DOCX 34 KB)

## Data Availability

Data will be made available upon request.
