# Supplementary material for: Affordable Care Act Medicaid expansion, access to health care, and financial behavior of the United States adults
Source: J Public Health Policy. 2024 Sep 23;45(4):740–56. doi: 10.1057/s41271-024-00522-0 (PMC11609092; doi:10.1057/s41271-024-00522-0)
Supplement: Supplementary file 1 — Supplementary file1 (DOCX 34 KB) [file 41271_2024_522_MOESM1_ESM.docx]

**Table S1: Descriptive Statistics of the Study Analytical Sample of Adults 45-64 below 100% FPL, 2009-2018 NCFS**

| **Variables** | **Weighted Percent or Mean** |
| --- | --- |
| **Age ^1^** | |
| 45-54 % | 55.66 |
| 55-64 % | 44.34 |
| **Sex ^1^** | |
| Male % | 42.23 |
| Female % | 57.77 |
| **Race ^1^** | |
| Non-Hispanic White % | 70.20 |
| Non-White % | 29.80 |
| **Education ^1^** | |
| Did not complete high school % | 11.26 |
| High school graduate % | 37.89 |
| Some college % | 34.71 |
| College graduate % | 14.05 |
| Postgraduate education % | 2.08 |
| **Marital status ^1^** | |
| Married % | 21.19 |
| Single % | 33.51 |
| Separated % | 5.24 |
| Divorced % | 32.46 |
| Widowed/widower % | 7.60 |
| **No. of children at home ^1^** | |
| None % | 73.10 |
| >=1 % | 26.90 |
| **Unemployed or Temporarily Laid Off Status ^1^** | |
| Yes % | 22.19 |
| No % | 77.81 |
| **Home Ownership ^1^** | |
| Yes % | 32.04 |
| No % | 67.96 |
| **Access to Care outcomes** | |
| Health Insurance Coverage ^1^ % | 67.02 |
| Unpaid Medical Bills ^1^ % | 35.48 |
| **Financial Outcomes** | |
| **Credit Card** | |
| Number of credit cards >=1 | 71.22 |
| Paid credit cards in full ^1^ % | 30.59 |
| **Bank** | |
| Has a checking account ^1^ % | 93.37 |
| Has a savings account, money market account, or CDs ^1^ % | 60.20 |
| Has investments in stocks, bonds, mutual funds, or other securities ^1^ % | 14.38 |
| **Financial Preparedness** | |
| Satisfied with your current personal financial condition (Mean) | 3.77 |
| Set aside emergency or rainy-day funds ^1^ % | 24.35 |
| Tried to figure out how much is needed to save for retirement ^1^ % | 20.24 |

Note: ^1^ The descriptive statistics of the variables are based on the regression estimation sample for health insurance as the dependent variable. FPL: Federal Poverty Level. NCFS: National Financial Capability Study.

**Table S2: State Assignment into Treatment and Control Groups**

| **Control states (Did not expand Medicaid under ACA by 2021)** | **Treatment states (Expanded Medicaid under ACA by 2021)** |
| --- | --- |
| Alabama  Florida  Georgia  Kansas  Mississippi  Missouri  Nebraska  North Carolina  Oklahoma  South Carolina  South Dakota  Tennessee  Texas  Wisconsin  Wyoming | Alaska ^a^  Arizona  Arkansas  California  Colorado  Connecticut  Delaware  District of Columbia  Hawaii  Illinois  Idaho ^d^  Indiana ^a^  Iowa  Kentucky  Louisiana ^b^  Maine ^c^  Maryland  Massachusetts  Michigan  Minnesota  Montana ^b^  Nevada  New Hampshire  New Jersey  New Mexico  New York  North Dakota  Ohio  Oregon  Pennsylvania ^a^  Rhode Island  Utah ^d^  Vermont  Virginia ^c^  Washington  West Virginia |

Note: All included states that expanded Medicaid under the ACA did so on Jan. 1^st^, 2014, except Michigan, which expanded in April 2014, and New Hampshire, which expanded in August 2014. Delaware, Massachusetts, New York, Vermont, and Washington, D. C. announced expansion under the ACA in 2014 but had full or near full expansions before 2014. Wisconsin expanded eligibility to 100% of the Federal Poverty Level in 2014 (not under the ACA). ^a^, ^b^, ^c^, and ^d^ indicate states that expanded Medicaid in 2015, 2016, 2019, or 2020. States that expanded Medicaid for a few months in 2021 (the last year of our study) were considered to be in the control group, as the beneficiaries did not enjoy a full year of benefits. Such states are – Missouri, which began processing Medicaid applications in October 2021; Nebraska, which announced plans to offer benefits from October 1, 2021; and Oklahoma, which began coverage of enrollees on July 1, 2021. ACA: Affordable Care Act.

**Table S3: Difference-in-Differences Estimates of the ACA Medicaid Expansion** **Effects on the Likelihood of Health Care Access for Adults Aged 45–64 below 100% FPL under Alternative Samples, NFCS 2009-2018**

|  | **Effect of the ACA Medicaid Expansion 2014–2018** | | | | | |
| --- | --- | --- | --- | --- | --- | --- |
|  | **Main Model** | **Model Not using Survey Sampling Weights** | **Model for 100-138% FPL** | **Model for 138-400% FPL** | **Model for**  **>400% FPL** | **Model**  **Including 2021** |
| Health Insurance Coverage | **0.127*** (0.033)** | **0.126*** (0.032)** | **0.134*** (0.029)** | **0.036*** (0.011)** | -0.008 (0.008) | **0.142^***^**  **(0.031)** |
| Unpaid Medical Bills | **-0.059* (0.034)** | **-0.061* (0.034)** | -0.041 (0.029) | 0.015 (0.016) | 0.024 (0.015) | -0.037  (0.028) |

Note: The models adjust for age, sex, race, education, marital status, children at home, employment status, and home ownership and include fixed effects for survey year and state. Standard errors are clustered by state and are presented in parentheses. The sample size ranges between 3,173 and 19,638, depending on the outcome. NFCS sampling weights are used. ^*^ *p* < 0.10, ^**^ *p* < 0.05, ^***^ *p* < 0.01. ACA: Affordable Care Act. NFCS: National Financial Capability Study. FPL: Federal Poverty Level.

**Table S4: Difference-in-Differences-in-Differences Estimates of the ACA Effects on the Likelihood of Financial Behaviors for Adults Aged 45–64 below 100% FPL under Alternative Samples, NFCS 2009-2018**

|  | **Effect of the ACA Medicaid Expansion 2014–2018** | | | | | |
| --- | --- | --- | --- | --- | --- | --- |
|  | **Main Model** | **Model Not using Survey Sampling Weights** | **Model for 100-138% FPL** | **Model for 138-400% FPL** | **Model for**  **>400% FPL** | **Model**  **Including 2021** |
| **Credit Card** | | | | | | |
| Number of credit cards | 0.009 (0.039) | 0.010 (0.037) | 0.021 (0.031) | 0.009 (0.012) | 0.011 (0.010) | -0.009  (0.034) |
| Paid credit cards in full | **0.086* (0.044)** | **0.092** (0.043)** | **0.056* (0.033)** | -0.034 (0.021) | 0.004 (0.019) | **0.070^*^**  **(0.040)** |
| **Bank** | | | | | | |
| Has a checking account | 0.025 (0.029) | 0.034 (0.028) | 0.012 (0.021) | 0.000 (0.009) | -0.007* (0.004) | 0.029  (0.026) |
| Has a savings account, money market account, or CDs | 0.018 (0.031) | 0.029 (0.031) | -0.006 (0.029) | -0.004 (0.015) | -0.000 (0.009) | 0.003  (0.028) |
| Has investments in stocks, bonds, mutual funds, or other securities | 0.003 (0.017) | 0.000 (0.018) | -0.019 (0.018) | 0.006 (0.016) | 0.018 (0.016) | 0.015  (0.019) |
| **Financial Preparedness** | | | | | | |
| Satisfied with your current personal financial condition | 0.120 (0.143) | 0.119 (0.145) | -0.134 (0.134) | 0.030 (0.111) | -0.015 (0.076) | 0.111  (0.140) |
| Set aside emergency or rainy-day funds | **0.048** (0.023)** | **0.049** (0.023)** | 0.016 (0.019) | -0.017 (0.014) | -0.007 (0.014) | **0.040^*^**  **(0.020)** |
| Tried to figure out how much is needed to save for retirement | 0.040 (0.032) | 0.047 (0.032) | 0.024 (0.029) | 0.012 (0.017) | -0.014 (0.016) | **0.050^*^**  **(0.027)** |

Note: The models adjust for age, sex, race, education, marital status, children at home, employment status, and home ownership and include fixed effects for survey year and state. Standard errors are clustered by state and are presented in parentheses. The sample size ranges between 1,657 and 19,579, depending on the outcome. NFCS sampling weights are used. * p < 0.10, ** p < 0.05, *** p < 0.01. ACA: Affordable Care Act. NFCS: National Financial Capability Study. FPL: Federal Poverty Level.

**Table S5: Pre-Trend Checks for Difference-in-Differences-in-Differences Estimates of the ACA Effects on Health Care Access for Adults Aged 45–64 below 100% FPL, NFCS 2009-2018**

|  | **Effect of the ACA Medicaid Expansion 2009** | **Effect of the ACA Medicaid Expansion 2012** | **P-value for joint test of Effect of the ACA Medicaid Expansion 2009 and 2012 coefficients** |
| --- | --- | --- | --- |
| Health Insurance Coverage | 0.031 (0.046) | 0.022 (0.057) | 0.8012 |
| Unpaid Medical Bills | 0.000 (.) | 0.021 (0.038) | 0.5768 |

Note: The models adjust for age, sex, race, education, marital status, children at home, employment status, and home ownership and include fixed effects for survey year and state. Standard errors are clustered by state and are presented in parentheses. The sample size ranges between 2,160 and 3,147, depending on the outcome. NFCS sampling weights are used. * p < 0.10, ** p < 0.05, *** p < 0.01. ACA: Affordable Care Act. NFCS: National Financial Capability Study. FPL: Federal Poverty Level.

**Table S6: Pre-Trend Checks for Difference-in-Differences Estimates of the ACA Medicaid Expansion Effects on Financial Outcomes for Adults Aged 45–64 below 100% FPL, NFCS 2009-2018**

|  | **Effect of the ACA Medicaid Expansion 2009** | **Effect of the ACA Medicaid Expansion 2012** | **P -value for joint test of Effect of the ACA Medicaid Expansion 2009 and 2012 coefficients** |
| --- | --- | --- | --- |
| **Credit Card** | | | |
| Number of credit cards | -0.043 (0.052) | -0.009 (0.045) | 0.7006 |
| Paid credit cards in full | -0.039 (0.073) | -0.015 (0.057) | 0.8564 |
| **Bank** | | | |
| Has a checking account | 0.045 (0.046) | -0.016 (0.035) | 0.3117 |
| Has a savings account, money market account, or CDs | -0.080 (0.052) | 0.009 (0.042) | 0.1451 |
| Has investments in stocks, bonds, mutual funds, or other securities | 0.010 (0.030) | 0.010 (0.024) | 0.9075 |
| **Financial Preparedness** | | | |
| Satisfied with your current personal financial condition | -0.185 (0.213) | -0.222 (0.227) | 0.5656 |
| Set aside emergency or rainy-day funds | 0.022 (0.035) | 0.036 (0.031) | 0.5266 |
| Tried to figure out how much is needed to save for retirement | -0.017 (0.040) | 0.009 (0.036) | 0.7600 |

Note: The models adjust for age, sex, race, education, marital status, children at home, employment status, and home ownership, including fixed effects for survey year and state. Standard errors are clustered by state and are presented in parentheses. The sample size ranges between 1,235 and 3,136, depending on the outcome. NFCS sampling weights are used. * p < 0.10, ** p < 0.05, *** p < 0.01. ACA: Affordable Care Act. NFCS: National Financial Capability Study. FPL: Federal Poverty Level.

**Table S7: Pre-Trend Checks for Difference-in-Differences Estimates of the ACA Medicaid Expansion Effects on Financial Outcomes for Adults Aged 45–64 below 100% FPL, NFCS 2009-2018**

| **Outcome** | **NFCS Question** |
| --- | --- |
| **Health Care Access** | |
| Health Insurance | Are you covered by health insurance? |
| Unpaid Medical Bills | Do you currently have any unpaid bills from a health care or medical service provider (e.g., a hospital, a doctor's office, or a testing lab) that are past due? |
| **Credit Card** | |
| Number of credit cards | How many credit cards do you have? |
| Paid credit cards in full | In the past 12 months, which of the following describes your experience with credit cards? - I always paid my credit cards in full |
| **Bank** | |
| Has a checking account | Do you [Does your household] have a checking account? |
| Has a savings account, money market account, or CDs | Do you [Does your household] have a savings account, money market account, or CDs? |
| Has investments in stocks, bonds, mutual funds, or other securities | Not including retirement accounts, do you [does your household] have any investments in stocks, bonds, mutual funds, or other securities? |
| **Financial Preparedness** | |
| Satisfied with your current personal financial condition | Overall, thinking of your assets, debts and savings, how satisfied are you with your current personal financial condition? |
| Set aside emergency or rainy-day funds | Have you set aside emergency or rainy day funds that would cover your expenses for 3 months, in case of sickness, job loss, economic downturn, or other emergencies? |
| Tried to figure out how much is needed to save for retirement | Have you ever tried to figure out how much you need to save for retirement? |

Note: The questions are from the NFCS codebooks 2009-2018. NFCS: National Financial Capability Study.
